# Implementation of strategies and programs for breastfeeding, complementary feeding, and malnutrition of young children in Brazil: advances and challenges

**DOI:** 10.1590/0102-311XEN053122

**Published:** 2023-10-20

**Authors:** Sonia Isoyama Venancio, Gabriela Buccini

**Affiliations:** 1 Instituto de Saúde, Secretaria de Estado da Saúde de São Paulo, São Paulo, Brasil.; 2 University of Nevada, Las Vegas, U.S.A.

**Keywords:** Breastfeeding, Complementary Feeding, Malnutrition, Nutrition Programs, Unified Health System, Aleitamento Materno, Alimentação Complementar, Desnutrição, Programas de Nutrição, Sistema Único de Saúde, Lactancia Materna, Alimentación Complementaria, Desnutrición, Programas de Nutrición, Sistema Único de Salud

## Abstract

Malnutrition in all its forms has risen on global agendas due to the recognition of its magnitude and consequences for a wide range of human, social, and economic outcomes. Implementing strategies and programs with the needed scale and quality is a major challenge. The *Brazilian National Survey on Child Nutrition* (ENANI-2019) pointed out several advances but numerous challenges. In this paper, we reflect on the implementation progress of breastfeeding, complementary feeding and young children malnutrition strategies and programs in Brazil and how existing challenges can be overcome through the lens of implementation science. First, we present a brief history of such programs. Second, we selected two breastfeeding initiatives to illustrate and reflect on common implementation challenges. In these case studies, we used the RE-AIM (Reach, Effectiveness, Adoption, Implementation, Maintenance) framework to analyze the implementation and scaling up barriers and facilitators. We found common barriers related to unclear goals about the reach of programs, challenges in assessing effectiveness and fidelity/quality during the real-world implementation, discontinuation or lack of funding, and lack of monitoring and evaluation impacting the sustainability of programs. We also discuss the use of implementation science to achieve adequate nutrition by 2030 and present critical elements for successful scale implementation of nutrition programs based on global evidence. Despite the investment to implement different actions aimed at facing infant feeding and malnutrition, high-quality implementation research must become a priority to catalyze progress in Brazil.

## Resumo

A desnutrição, em todas as suas formas, cresceu nas agendas globais devido ao reconhecimento de sua magnitude e consequências para uma ampla gama de resultados humanos, sociais e econômicos. Implementar estratégias e programas com a escala e a qualidade necessárias é um grande desafio. O *Estudo Nacional de Alimentação e Nutrição Infantil* (ENANI-2019) apontou vários avanços, mas inúmeros desafios. Este artigo reflete sobre o progresso da implementação de estratégias e programas de amamentação, alimentação complementar e desnutrição de crianças pequenas no Brasil e como os desafios existentes podem ser superados através das lentes da ciência da implementação. Primeiramente, um breve histórico de tais programas é apresentado. Em seguida, duas iniciativas de amamentação selecionadas para ilustrar e refletir sobre os desafios comuns de implementação. Nesses estudos de caso, o modelo RE-AIM (alcance, eficácia, adoção, implementação e manutenção) foi utilizado para analisar a implementação e ampliar barreiras e facilitadores. Foram encontradas barreiras comuns relacionadas a metas pouco claras sobre o alcance dos programas, desafios na avaliação da eficácia e fidelidade/qualidade durante a implementação no mundo real, a interrupção ou falta de financiamento e a falta de monitoramento e avaliação que afetam a sustentabilidade dos programas. O uso da ciência da implementação para alcançar uma nutrição adequada até 2030 também foi discutido, apresentando elementos críticos para a implementação em escala bem-sucedida de programas de nutrição com base em evidências globais. Apesar do investimento para implementar diferentes ações voltadas para o enfrentamento da alimentação infantil e da desnutrição, a pesquisa de implementação de alta qualidade deve se tornar uma prioridade para catalisar o progresso no Brasil.

## Resumen

La desnutrición, en todos sus grados, es un tema que está presente en las agendas mundiales debido a su magnitud y consecuencias para una amplia gama de resultados humanos, sociales y económicos. La implementación de estrategias y programas con la escala y calidad requeridas es un gran desafío. El *Estudio Nacional de Alimentación y Nutrición Infantil* (ENANI-2019) apunta varios avances y numerosos desafíos. Este artículo reflexiona sobre el progreso en la implementación de estrategias y programas para la lactancia materna, la alimentación complementaria y la desnutrición infantil en Brasil y sobre cómo se pueden superar los desafíos existentes desde la implementación. En primer lugar, se presenta una breve historia de tales programas. A continuación, se seleccionan dos iniciativas de lactancia materna para ilustrar y reflexionar sobre los desafíos comunes de implementación. En este estudio de caso se utilizó el modelo RE-AIM (alcance, eficacia, adopción, implementación y mantenimiento) para analizar la implementación y ampliar las barreras y facilitadores. Se encontraron barreras comunes relacionadas con los objetivos poco definidos sobre el alcance de los programas, con los desafíos para evaluar la efectividad y la fidelidad/calidad durante la implementación, con la interrupción o falta de financiamiento y con la falta de monitoreo y evaluación que afectan la sostenibilidad de los programas. También se discutió el uso de la implementación para lograr una nutrición adecuada para 2030, mediante la presentación de elementos críticos para implementar con éxito los programas de nutrición basados en evidencia global. Si bien se promueve la implementación de diferentes acciones destinadas al combate de la alimentación infantil y la desnutrición, la implementación de gran calidad debe ser una prioridad en Brasil.

## Background

The increase in overweight concurrent with persistent undernutrition in young children has led to the double burden of malnutrition ^1^ ^,^ ^2^. In recognition of the magnitude and the health, social, and economic consequences of malnutrition in all its forms, the United Nations Decade of Action on Nutrition (2016-2025) and the 2030 Sustainable Development Goals (SDGs) are among the many global examples that have embraced young children’s nutrition and development as key to the social transformation desired globally ^3^.

The Nurturing Care Framework outlines five components to strengthen policies and systems to ensure that all children reach their development potential ^3^. Among these components, “adequate nutrition” from pregnancy through early childhood plays a powerful role in enabling a child to grow, learn, and thrive ^3^ ^,^ ^4^. Young children flourish with exclusive breastfeeding - from immediately after birth to 6 months of age. At about 6 months of age ^5^, in addition to breastmilk, young children need complementary foods that should be offered in a responsive way, be frequent and diverse, and provide the micronutrients needed for the rapid growth of their body and brain ^5^ ^,^ ^6^.

Despite the many evidence-based interventions available to support young children nutrition ^4^, only about one-third of countries are on track to achieve the global stunting target, and roughly one-half are on track for the wasting and exclusive breastfeeding targets ^7^. Therefore, implementing at scale policies, programs, and interventions with the quality needed to achieve global impact is a major challenge.

The *Brazilian National Survey on Child Nutrition* (ENANI-2019) provided information on nutritional status, infant and young children feeding practices, and micronutrient deficiencies, indicating several advances but numerous challenges that remain to achieve the 2030 SDGs. Attention is drawn, for example, to the increased consumption of ultra-processed foods in the first years of life ^8^. Thus, the main objectives of this article are to: (1) describe the history of infant and young children nutrition strategies and programs at the national level in Brazil; (2) present an implementation analysis of two breastfeeding strategies; (3) discuss the critical role of implementation science research for scale up and sustainability of infant and young children nutrition programs; and (4) summarize lessons learned and policy implications for Brazil.

## A brief history of infant and young nutrition strategies and programs in Brazil

### The Brazilian legal framework for infant and young children food and nutrition programs

**Figure 1:**
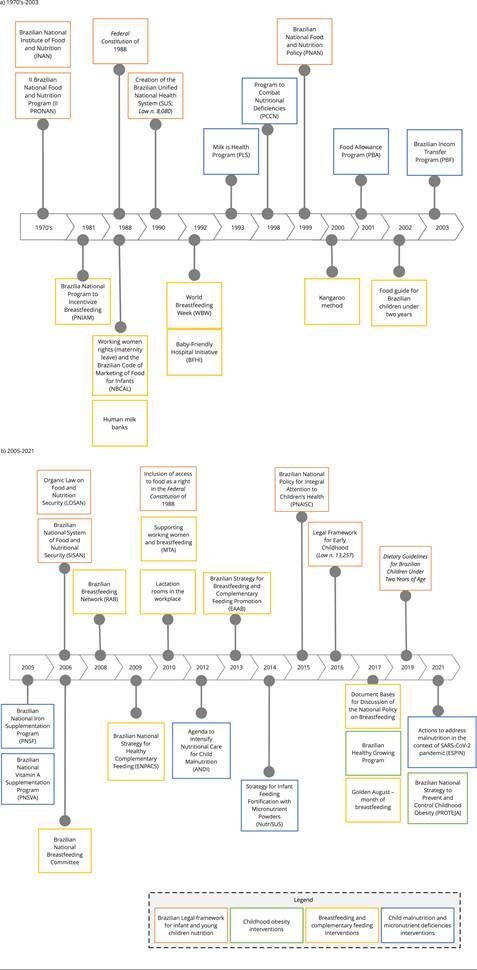
Timeline of infant and young children nutrition-related policies, strategies, and actions in Brazil.

Figure 1 summarizes a timeline of the policy environment for infant and young children nutrition in Brazil. The timeline starts in the early 1970s, during the military dictatorship, with the creation of the Brazilian National Institute of Food and Nutrition (INAN), a public autarchy linked to the Brazilian Ministry of Health. The II Brazilian National Food and Nutrition Program (II PRONAN) began in 1976 to integrate food production and distribution with food supplementation assistance. The II PRONAN encompassed nine programs, several of which included aspects aimed at children, such as the Brazilian National Nutrition in Health Program, the Program to Combat Specific Nutritional Deficiencies (PCCNE), the Brazilian National Food and Nutrition Surveillance System (SISVAN), the Brazilian National School Meal Program (PNAE), and the Brazilian National Program to Promote Breastfeeding (PNIAM) ^9^.

ESPIN: Public Health Emergency of National Importance.

In the 1980s, social movements focused on defending the return to democracy generated the Brazilian Health Reform Movement. The *Federal Constitution* of 1988 incorporated a concept of social security as an expression of the social rights inherent to citizenship through the guarantee of a set of economic and social policies, including creating the Brazilian Unified National Health System (SUS) ^10^. Adequate nutrition was recognized as a determining and conditioning factor for the health of individuals and communities. Thus, the Organic Health Law (*Law n. 8,080*), which established the SUS, provided guidance for monitoring nutritional and dietary practices.

At the end of the 1990s, the Brazilian National Food and Nutrition Policy (PNAN) was created and coordinated by the Brazilian Ministry of Health through the General Coordination of Food and Nutrition Policy (CGPAN), currently the General-Coordination of Food and Nutrition (CGAN) ^11^. Following the publication of the PNAN, a set of political and legal frameworks in the field of food and nutrition security was approved, such as the Organic Law on Food and Nutrition Security (LOSAN) and the Brazilian National System for Food and Nutrition Security (SISAN) ^9^. Between 2010 and 2011, PNAN was revised to incorporate the interlocution between the SUS and SISAN as well as guide the organization and qualification of interventions related to food and nutrition in the context of healthcare networks ^9^.

In this period, along with important achievements of the SUS, such as the expansion of primary health care coverage, the reduction of infant mortality rates, and the promotion of food and nutrition security, Brazil experienced several changes in socioeconomic and demographic indicators that led to an epidemiological and nutritional transition ^12^.

After the extinction of INAN in 1997, breastfeeding promotion, protection, and support were coordinated by the children’s health area within the Brazilian Ministry of Health. In 2015, the Brazilian National Policy for Integral Attention to Children’s Health (PNAISC) posed a set of comprehensive actions and strategies across seven pillars to promote child development and mitigate vulnerabilities (*Ordinance GM/MS n. 1,130* of August 5, 2015). Specifically, the second pillar explicitly promotes breastfeeding and healthy complementary feeding. 

Finally, the Legal Framework for Early Childhood was published (*Law n. 13,257*, of March 8, 2016), establishing principles and guidelines for formulating public policies to fulfill the rights of children, increase the effectiveness of integrated policies, define strategies for intersectoral coordination, and define food and nutrition as priority areas.

### The Brazilian infant and young children food and nutrition strategies and programs in the health sector

We organized into three themes a brief historical overview of strategies and programs on children’s food and nutrition implemented in the health sector in Brazil: (1) child malnutrition and micronutrient deficiencies; (2) breastfeeding and complementary feeding; and (3) childhood obesity. A detailed description of these strategies and programs is presented in Supplementary Material (https://cadernos.ensp.fiocruz.br/static//arquivo/supl-e00053122_5122.pdf). 

#### Child malnutrition and micronutrient deficiencies

The 2013 *Lancet Series* on maternal and child nutrition prioritized ten interventions that, if scaled to 90% coverage in 34 high-burden countries, could reduce child mortality by 15% and stunting by about 20%. Nine of the ten interventions are usually delivered through health systems ^13^. Recent systematic reviews and meta-analyses recommended prioritizing newborn interventions, including delayed cord clamp, skin-to-skin contact, breastfeeding within the first hour, promotion of breastfeeding and age-appropriate complementary feeding, and micronutrient interventions for children at risk ^14^. The latter focuses on high-dose vitamin A supplementation, micronutrient powders used for point-of-use fortification of complementary foods to prevent anemia, preventive zinc supplementation, and wasting prevention and treatment ^14^.

(a) Child malnutrition. Substantial declines in the prevalence of child undernutrition in Brazil have been attributed to gains in family income, maternal schooling, and expanded coverage of public education, sanitation, and health care services ^12^. Initially, food supplementation programs with milk distribution consisted in one pillar for fighting hunger and maternal and child malnutrition, however over the decades, Brazil expanded the nature of actions in the health sector to combat malnutrition ^15^.

Over time, these programs have incorporated new approaches and been replaced by more comprehensive actions. The II PRONAN, in the late 1970s, announced a set of activities with a broader approach to combat hunger and malnutrition. However, many of these actions never came to fruition. The “Milk is Health Program” (PLS), launched in 1993, is one example of a food supplementation program. The goal of PLS was to reduce the prevalence of malnutrition by providing free milk supplementation for specific groups (including children under five years of age) identified through the SISVAN ^16^, which was institutionalized as a responsibility of the Brazilian Ministry of Health in the early 1990s. Despite the biological and palliative focus of the PLS, it aimed to reinforce the provision of primary health actions and contribute to the implementation of the SUS, specifically the municipalization and reorganization of services. Although nutrition surveillance was not mandatory in the municipalities, it was expected that the integration of SISVAN into the PLS would catalyze the implementation of nutrition surveillance activity ^17^. However, a study conducted in Bahia found that PLS did not guarantee SISVAN implementation, because the local nutritional epidemiological profile was not used to address food and nutrition problems ^17^. 

In the late 1990s, the Program to Combat Nutritional Deficiencies (PCCN) was created to replace the PLS. The PCCN included actions beyond food supplementation, such as promoting breastfeeding, monitoring nutritional status, and preventing and treating iron and vitamin A deficiencies. The estimates of the target audience for PCCN were based on statistical models prepared by the Center for Nutrition and Health Research, University of São Paulo (NUPENS/USP), which significantly changed the transfer of federal resources to the fight malnutrition, reversing traditional practices of equal treatment for unequal situations ^18^.

In the 2000s, food supplementation programs were replaced with cash transfer programs, which had more significant potential for addressing the social determinants of malnutrition ^15^. Cash transfer programs are widely implemented to alleviate poverty and provide safety nets to vulnerable households with children ^19^. The first of these initiatives to improve nutrition was the Food Schoolarship Program under the health sector coordination. The Food Schoolarship Program defined biological vulnerability as an inclusion criterion, i.e., malnutrition in pregnant and lactating women or children between six months and six years old. In addition to financial support for low-income families facing nutritional insecurity, the Food Schoolarship Program encouraged their participation in primary health actions.

In 2003, the Food Schoolarship Program was replaced by the Brazilian Income Transfer Porgram, in which social vulnerability was prioritized, defining a family poverty cutoff as the criterion for eligibility accompanied by health and education conditionalities. The health conditionality included a minimum of health care visits for children of beneficiaries’ families and monitoring the nutritional status. Evidence of programs to prevent and control malnutrition shows that positive responses to child malnutrition are closely related to confronting social determinants of health and equity-oriented policies, i.e., the redistribution of income and guarantee of universal access to health, education, and basic sanitation ^15^. Despite solid evidence on the impact of the Brazilian Income Transfer Porgram, during its 18 years of implementation, on infant and maternal mortality ^20^ ^,^ ^21^ ^,^ ^22^ and outcomes related to food and nutrition ^23^ ^,^ ^24^ ^,^ ^25^ ^,^ ^26^, it was discontinued in October 2021 and replaced by Brazil Assistance Program, a new CTP with a different scope of work and eligibility criteria. This replacement brings concerns and uncertainties about the continued impact of cash transfer programs on children’s nutritional and health outcomes in Brazil.

Despite reducing the prevalence of malnutrition in Brazil, the problem persists in some population subgroups, demanding focused attention and social investments. In 2012, the Brazilian Ministry of Health instituted the Agenda to Intensify Nutritional Care for Child Malnutrition targeting Brazilian municipalities with the highest prevalence of malnutrition among children under five based on data from SISVAN. However, this strategy was only implemented and financed from 2012 to 2015 ^9^. The COVID-19 pandemic has crippled the health system and reversed economic growth in Brazil, potentially setting back malnutrition improvements. The pandemic has also exacerbated food insecurity among households with children ^27^. This context led to the need for an urgent organization of nutrition and FNS actions within the scope of primary care, focusing on children and pregnant women. Specifically, the Public Health Emergency of National Importance (ESPIN) declaration provided financial incentives within the scope of actions combating malnutrition to strengthen the care for children in the states and municipalities (Chapter III of *Ordinance GM/MS n. 894/2021*).

(b) Micronutrients deficiencies. The actions to combat micronutrient deficiencies in the health sector were initially included in programs to combat malnutrition ^28^. However, since the 2000s, specific programs have been launched, including the Brazilian National Iron Supplementation Program (PNSF), the Brazilian National Vitamin A Supplementation Program (PNSVA), and the Brazilian National Strategy to Fortification Infant Feeding with Micronutrients Powder (NutriSUS) ^28^. The PNSF included preventive iron supplementation for children, pregnant and postpartum women, as well as those who miscarry, implemented by the primary health teams. It also had the mandatory fortification of wheat and corn flour with iron and folic acid as well as nutritional guidance. The PNSF was updated in 2013 when the purchase of ferrous sulfate became decentralized to the municipalities. The target audience was expanded to include children aged 6 and 24 months, pregnant women starting prenatal care, and postpartum women. The administration of ferrous sulfate became daily for children. Brazil pioneered vitamin A in national immunization campaigns, a strategy later advocated by the World Health Organization (WHO) and the United Nations Children’s Fund (UNICEF). Since 1983, the Brazilian Ministry of Health has used megadose of vitamin A ^29^. The PNSVA began in 2005 and initially included children from the Northeastern regions and municipalities of Vale do Jequitinhonha and Mucuri in Minas Gerais State. In 2013, the PNSVA was expanded to all municipalities in the Northern region, municipalities that are part of Brazil without Extreme Poverty Plan in the Central-western, Southern, and Southeastern regions of the country, and all Special Indigenous Sanitary Districts (DSEIs) ^28^. In 2014, the NutriSUS began to add a mixture of powdered vitamins and minerals to one of the daily meals offered to 6-48-month-olds in daycare centers. Before formulating this strategy, a multicenter study in four Brazilian municipalities was conducted to evaluate its effectiveness and found positive results in reducing the prevalence of anemia, vitamin A deficiency, and iron deficiency. NutriSUS has been scaled up to several Brazilian municipalities and 20 DSEIs ^30^. Currently, objectives, target audience, and implementation strategies of micronutrient supplementation programs were reviewed by CGAN, involving specialists in the field, researchers, and managers, using the most up-to-date evidence provided by ENANI-2019 ^31^. 

#### Breastfeeding and complementary feeding

At the end of the 1970s, following the international movement led by the WHO and UNICEF to return to breastfeeding, the II PRONAN included a proposal to create a program to promote breastfeeding. However, this proposal was only put into effect in 1981, creating the PNIAM. The PNIAM gained international attention for its diversity of actions aimed at promoting (e.g., advertising campaigns broadcast by the mass media), protecting (e.g., labor laws to protect breastfeeding and regulations from controlling marketing and commercialization of artificial milk), and supporting breastfeeding (e.g., breastfeeding support groups in the community, and individual counseling) ^32^. In 1983, a crucial step was taken related to maternity hospital practices, with the publication of the Joint Rooming-in Ordinance, making it mandatory for the baby to stay with the mother full-time in public hospitals ^33^. Brazil was a pioneer in recommending this practice that years later was incorporated into the Baby-Friendly Hospital Initiative (BFHI). 

The enactment of the *Federal Constitution* and the formation of the SUS in 1988 impacted children’s healthcare in Brazil, evolving from vertical maternal-infant programs of the 1970s and 1980s to the perspective of comprehensive care, aimed at ensuring rights, overcoming vulnerabilities, reducing morbidity and mortality, and promoting health and quality of life ^34^. The *Federal Constitution* guaranteed to working women, with a formal employment relationship, benefits including 120 days of maternity leave, the right to two half-hour breaks during the workday to breastfeed the child up to six months of age, and the right to daycare in the workplace. In 2008, *Law n. 11,770* established the Corporate Citizen Program, aimed at extending maternity leave to six-month by granting a tax incentive. In addition, the Supporting Working Women and Breastfeeding (MTA) was adopted in partnership with the Brazilian Society of Pediatrics. It consisted of three strategic axes: extension of maternity leave to 180 days, implementation of daycare in the workplace, and creation of lactation rooms in the workplace ^33^.

Another milestone for legal protection was created in 1988 with the Brazilian Code of Marketing of Food for Infants (NBCAL) ^33^ ^,^ ^35^, which aimed to protect breastfeeding by prohibiting the advertising of food products to children, providing free samples to mothers, promoting these products in health services, and providing gifts and samples to healthcare workers. The NBCAL was based on the International Code of Breastmilk Substitutes, proposed by the WHO in 1981, and developed by a working group established by the Brazilian Ministry of Health with prominent role of the International Baby Food Action Network (IBFAN). This legislation has undergone numerous revisions over the years, with the publication of several ministerial ordinances, resolutions, laws, and decrees ^35^. 

In 1988, the Brazilian Ministry of Health also regulated the human milk banks as centers for collecting, processing, and storing human milk and proving skilled lactation support ^33^. In the late 1990s, ministerial ordinances were published creating the Brazilian National Commission of Human Milk Banks and the Brazilian Network of Human Milk Banks under the Oswaldo Cruz Foundation (Fiocruz) reference center ^33^. The Brazilian human milk bank model has been recognized worldwide for its technological development, which combines low cost with high quality. Since then, the Brazilian Network of Human Milk Banks has established international cooperation with several countries and the Global Network of Human Milk Banks.

In the early 1990s, Brazil was one of the twelve countries to adopt the BFHI. To leverage its implementation, the Brazilian Ministry of Health intensified the availability of four courses proposed by the WHO: an 18-hour course for maternity teams; an 80-hour course to train monitors; a 40-hour Breastfeeding Counseling Course; and Quick Course aimed at raising awareness among managers ^36^. The Brazilian BFHI underwent revisions and, in addition to complying with the Ten Steps to Successful Breastfeeding, to be certified, a hospital has to comply with the NBCAL, the mother-friendly care, and must ensure that the mother or father (or legal guardian) remains with the newborn 24-hours. In the same period, Brazil began to celebrate World Breastfeeding Week (WBW) in the first week of August, which has become an important strategy to promote breastfeeding. In 1999, the Brazilian Ministry of Health took over the coordination of WBW and became responsible for adapting the theme proposed by the World Alliance for Breastfeeding Action (WABA) in the country as well as creating and distributing posters and folders. *Law n. 13,435/2017* established August as the Month of Breastfeeding - “Golden August” ^36^.

The 2000s marked important advances to protect, promote, and support breastfeeding. The Kangaroo method, is codified in the Standard for Humanized Attention of Low-Birth Weight Newborns ^33^. It started as a hospital-based initiative and showed to be effective to increase breastfeeding rates, among other countless benefits ^37^ ^,^ ^38^. The implementation manual of 2018 made advances towards the organization of shared care between hospital and primary care teams ^39^. 

The promotion of healthy complementary feeding was intensified by developing and disseminating various educational materials targeting health professionals. These include the *Dietary Guidelines for Brazilian Children Under Two Years of Age* (published in 2002 and revised in 2010). These “dietary guidelines” were revised again in 2019 to respond to recent changes in social transformations and dietary practices and align its approach and recommendations with the dietary guidelines for the Brazilian adult population ^40^.

To strengthen the governance of breastfeeding actions in 2006, the Brazilian National Breastfeeding Committee was created. In 2012, the composition of this national committee was revised to include a representation of a mothers’ group, civil society, international organizations, and representatives of educational institutions ^33^. The committee was deactivated under *Decree n. 9,759* of April 11, 2019, and was reactivated under *Ordinance GAB/SAPS n. 13*, 2022 as a Technical Advisory Council.

The Brazilian Breastfeeding Network (RAB) was created in 2008 and the Brazilian National Strategy for Healthy Complementary Feeding (ENPACS) in 2010, both based on the principles of critical-reflexive education and the SUS Continuing Health Education Policy, which aimed to review and support interdisciplinary work processes in primary health units to increase the prevalence of breastfeeding and healthy complementary feeding ^41^. Given the operational difficulties in implementing the RAB and ENPACS, in 2013, the two initiatives were integrated into the Brazilian Strategy for Breastfeeding and Complementary Feeding Promotion (EAAB). The EAAB uses the theoretical references of critical-reflective education and aims to facilitate the participation of primary health professionals in continuing education workshops to enhance their work in promoting breastfeeding and complementary feeding ^42^.

An analysis of the implementation of actions to promote, protect, and support breastfeeding concluded that the process which led to the successful scaling up of breastfeeding promotion in Brazil included investments in the following areas: (1) baseline needs assessment including data on infant feeding practices; (2) advocacy (including using scientific evidence to educate decision-makers about the health and economic benefits of breastfeeding and international consensus on breastfeeding policies/recommendations); (3) national and local mass media campaigns, social mobilization (e.g., WBW); (4) implementation and spread of the BFHI; (5) lactation management and communications/counseling training (development of human resources); (6) legislation (maternity/paternity leave, breastfeeding at work); and (7) monitoring and evaluation (including monitoring of the WHO code) ^43^. Rollins et al. ^44^ point to Brazil as an example of a country in which policies and programs address the three levels of the conceptual model that determine breastfeeding (individual, scenarios, and structural) implemented simultaneously, with visible leadership, government investments, and active participation of civil society. Despite advances, in 2017, the document *Bases for Discussion of the National Policy on Breastfeeding* ^33^ highlights the implementation of a Brazilian National Policy on Breastfeeding across inter-federative bodies and with the health sector coordinating multisectoral actions could be a way to guarantee advances in breastfeeding.

#### Childhood obesity

In 2011, the PNAN expanded the concept of healthy eating and comprehensive care for diseases related to food and nutrition, such as childhood obesity. It defined a set of actions within the health sector and other sectors to ensure environments that favor healthy eating and active lifestyles. In the same year, the Federal Government launched the Strategic Action Plan to Combat Chronic Noncommunicable Diseases (2012-2022), recognizing obesity as a disease and a risk factor for noncommunicable diseases. In 2014, the Interministerial Committee on Food and Nutrition Security (CAISAN), an intersectoral committee to articulate the ministries for the food and nutrition security agenda, launched the document Intersectoral Strategy for the Prevention and Control of Obesity. 

Two initiatives that stand out to combat childhood obesity specifically were the Brazilian Healthy Growing Program (2017) ^45^ and the Brazilian National Strategy to Prevent and Control Childhood Obesity (PROTEJA) ^46^. The Brazilian Healthy Growing Program, created in 2017, establishes, within the scope of the Brazilian School Health Program (PSE), a set of actions to help combat childhood obesity in the country through actions within the scope of the PSE, for children enrolled in early childhood education (daycare and preschools) and elementary school. PROTEJA aims to reduce childhood obesity and improve the health and nutrition of Brazilian children. This strategy of the Brazilian Ministry of Health pushed managers, health professionals, civil society, and partners to recognize childhood obesity as a priority public health problem and share responsibility in implementing effective measures to prevent and focus on childhood obesity in the country. PROTEJA contemplates a set of essential and complementary actions implemented together at the municipal level to help prevent and reduce childhood obesity ^46^.

Importantly, both Brazilian actions to fight against child obesity are aligned to the recommendations published by the WHO Commission on Ending Childhood Obesity in 2016, which states that “*obesity prevention and treatment requires a whole-of-government approach in which policies across all sectors systematically take health into account, avoid harmful health impacts, and thus improve population health and health equity*” ^47^.

## An implementation science analysis of breastfeeding policies

Brazil’s long trajectory builds a robust framework for infant and young children’s nutrition policies and initiatives; however, there are still challenges for their implementation. We selected breastfeeding protection, promotion, and support initiatives - the BFHI (focused on hospital settings) and the EAAB (focused on primary health settings) - to illustrate and reflect on common implementation challenges. In these case studies, we used the RE-AIM (Reach, Effectiveness, Adoption, Implementation, Maintenance) framework to analyze the implementation and scaling up barriers and facilitators ^48^. The RE-AIM is a well-known implementation science framework that can guide the selection, adaptation, and evaluation of interventions on key dimensions associated with successful implementation ^48^. The RE-AIM framework helps define whose health or health behavior will benefit from the intervention (Reach), identify which components of the intervention are considered the “active ingredients” necessary for the desired impact (Effectiveness); describe relevant characteristics of the delivery setting and those involved in delivering the intervention (Adoption); evaluate the extent that the active ingredients are delivered with fidelity to the established protocols (Implementation); and describe facilitators and barriers that may influence organizational decisions to sustain the intervention after the study is completed (Maintenance) ^49^. 

### Case study 1: the Brazilian Baby-Friendly Hospital Initiative

The BFHI was conceived in the early 1990s by the WHO and UNICEF, ratified by the *Innocenti Declaration* and the World Health Assembly resolutions of 1994 and 1996, and in 2002 included in the Global Strategy for Infant and Young Child Feeding ^50^. The BFHI was revised in 2004-2005 and again in 2018, in which the essence of the initially proposed Ten Steps to Successful Breastfeeding (“Ten Steps”) was maintained, but the wording of each step was updated in line with the current scientific evidence-based guidelines globally ^51^. Robust scientific evidence has been collected on the effectiveness of the BFHI ^52^ ^,^ ^53^ ^,^ ^54^ ^,^ ^55^ ^,^ ^56^; however, several implementation challenges have been identified worldwide related to its coverage, designation, and discreditation ^53^.

The Brazilian BFHI accreditation follows the global criteria to comply with the “Ten Steps” and three additional criteria: (1) compliance with the NBCAL, (2) the mother-friendly care practices, and (3) assurance that the mother or father (or legal guardian) remains with the newborn 24-hours. In 2022, the Brazilian BFHI will complete 30 years of implementation. However, despite considerable efforts made by the Brazilian Ministry of Health as well as State and Municipal Health Departments to mobilize managers and professionals from maternity hospitals to adopt the Ten Steps, its implementation at scale is still facing several challenges. Box 1 presents our analysis following the RE-AIM dimensions.

**Box 1:** Implementation scenario of the Baby-Friendly Hospital Initiative (BFHI) and Brazilian Strategy for Breastfeeding and Complementary Feeding Promotion (EAAB) based on the dimensions of the RE-AIM framework.

DIMENSIONS OF RE-AIM	DEFINITION	BFHI SCENARIO	EAAB SCENARIO
Reach	The absolute number, proportion, and representativeness of individuals willing to participate in a given initiative, intervention, or program.	About 25% of the total number of live hospital births in Brazil occur in hospitals-accredited as baby-friendly ^52^. Of the 3 million births occurring in Brazil per year, 98% occur in hospitals, which is a window of opportunity for all children to benefit from the practices recommended by the BFHI.	The reach of the EAAB has not been monitored during the implementation; therefore, the percentage of the target population reached by the strategy is unknown.
Effectiveness	The impact of an intervention on important outcomes.	Despite the low reach of the Brazilian BFHI, several national studies point to the impact of the BFHI on indicators such as breastfeeding in the first hour of life, exclusive breastfeeding for the first six months, reduction of pacifier use, and consequent increase of exclusive breastfeeding duration ^52^ ^,^ ^53^ ^,^ ^54^ ^,^ ^55^. Therefore, the Brazilian BFHI is an example of difficulties incorporating evidence into health services.	One important challenge to measure EAAB’s effectiveness is the difficulty of health professionals in monitoring breastfeeding and complementary feeding indicators. Although Brazil has the SISVAN, the coverage of monitoring children 0 to 2 years old was only 5.1% in the 382 municipalities that had greater adherence to the EAAB. Thus, only a few studies have analyzed the impact of the EAAB on breastfeeding and complementary feeding indicators. A study conducted to assess the effectiveness of using a manual to support the tutor implementing the EAAB found a positive impact on complementary feeding indicators, such as minimal food diversity and adequacy of children’s diet, and no effect on exclusive breastfeeding in children under six months ^58^. Therefore, more studies need to be conducted to fill this important gap, about whether EAAB can impact infant and young children’s nutritional outcomes.
Adoption	The absolute number, proportion, and representativeness of: (a) settings and (b) intervention agents (people who deliver the program) who are willing to initiate a program.	Between 1992 and 2010, 322 hospitals were accredited in the BFHI. However, after 2005, a deacceleration of the number of qualifying hospitals was observed, and others were discredited. In 2021, Brazil had only 302 accredited-hospitals ^52^. Although present in all 26 Brazilian states and the Federal District, most accredited hospitals are in the Northeastern Region (111), followed by the Southeastern Region (78), the Southern Region (55), the Central-Western Region (34), and the lowest number in the Northern Region (24), which points to important regional inequalities ^52^. In addition, less than 10% of hospitals with maternity wards in the country are accredited in the BFHI. There is a consensus among policymakers, managers, health professionals, and researchers that the addition of the other criteria, in addition to those established globally, has hindered the adoption and scale-up of the BFHI in Brazil ^52^. The process of accreditation of hospitals by the BFHI has not been uniform, with variations depending on the support of state and municipal authorities and hospital management, in particular the availability of financial resources for the entire process of qualifying the hospital ^52^.	According to data from the EAAB Management System, from 2013 until 2019, 5,959 tutors were trained, 3,290 primary health teams/clinics received workshops, and 48,640 primary health professionals participated in training activities ^59^. However, according to the EAAB monitoring system only 382 of the 5,575 Brazilian municipalities registered workshops between 2015 and 2019. In addition, considering the number of existing primary health teams/clinics and the number of tutors trained, the possible coverage of the EAAB in the country is 9.42%, with the Central-Western and Northern regions exhibiting the greatest possibility of coverage and the Northeastern Region having the least possibility to cover the primary health teams/clinics ^59^.
Implementation	At the setting level, implementation focuses on fidelity to an intervention, which is the extent to which the program is implemented consistently across different settings, staff, and patients. It also includes adaptations made and costs from multiple stakeholder perspectives.	Special attention must be paid to BFHI accreditation, i.e., fidelity based on compliance with the “Ten Steps” and the additional three core criteria. The assessment of compliance with the criteria has been evaluated through a complex system established by the Brazilian Ministry of Health, which includes the evaluation stages for accreditation and triennial reassessments, both conducted by external trained evaluators, and annual self-monitoring of maternity hospitals. The self-monitoring conducted between 2010 and 2015 by 143 accredited hospitals observed greater than 70% adherence for most criteria ^50^. However, the external reassessments conducted in 2015 found a lower percentage of compliance with some criteria, such as mother-friendly care and breastfeeding in the first hour of life (Step 4), both below 50% ^50^. The same was identified in the *Birth in Brazil* research, in which breastfeeding at birth was considered low, even in accredited-BFHI hospitals (24% offered breast at delivery room and 56% were breastfed within the first- hour) ^56^. Another implementation barrier is related to the budget availability of the Brazilian Ministry of Health to reimburse practices within the Brazilian BFHI; for example, accredited hospitals should receive 17% reimbursement for a vaginal delivery or 8.5% for a cesarean ^52^. In addition, the lack of financial resources to support the travel of the BFHI trained evaluators is a barrier to conducting the assessment, which can impact the quality of the BFHI program.	Analysis of fidelity is an important challenge of the EAAB. Based on the certification criteria, from 2015 to 2019, only 189 primary health teams/clinics had achieved all the criteria, representing 1.8% of the total primary health teams/clinics of the 382 municipalities that registered any continuing education workshops in that same period (data from EAAB monitoring system) An additional challenge is that the EAAB does not define a priori intervention model (such as conducting groups or home visits), the time of the intervention (prenatal, postnatal period, or both), the professionals who must be involved in its delivery or its intensity, which makes the analysis of fidelity complex. On the one hand, there is greater flexibility for adaptations to local contexts; on the other hand, teams have not been supported to design their interventions based on evidence, which can compromise the quality of interventions when they are developed. Another barrier to implementation of the EAAB is the lack of financial resources from the Brazilian Ministry of Health to support its implementation ^57^. In 2013, the fund-to-fund transfer for food and nutrition interventions was revised and was an important advance to support the implementation of EAAB in municipalities with more than 150,000 inhabitants. However, state and municipal managers find it difficult to use these funds, and many times they have not been used ^74^. Through *Ordinance n. 3,297/2020*, for the first time, the Brazilian Ministry of Health transferred resources to municipalities that had started holding EAAB workshops. A third barrier of EAAB implementation is the lack of institutionalization as municipalities can choose whether or not they want to roll out EAAB. Therefore, political will is critical to the implementation of EAAB at the municipal level ^57^.
Maintenance	The extent to which: (a) behavior is sustained six months or more after treatment or intervention; and (b) a program or policy becomes institutionalized or part of the routine organizational practices and policies. Includes proportion and representativeness of settings that continue the intervention and reasons for maintenance, discontinuance, or adaptation.	The sustainability of BFHI in Brazil is a critical aspect since 23 hospitals were discredited as of 2005 ^52^. In addition, the percentages of noncompliance with the Ten Steps and additional criteria identified in the self-monitoring and external reassessments indicate difficulties in the institutionalization of the evidence-based practices recommended by the BFHI in the routine of maternity hospitals ^52^.	Unfortunately, after EAAB certification, the primary health teams/clinics have not been monitored. Therefore, without improving mechanisms of monitoring and quality, the sustainability of EAAB is uncertain.

SISVAN: Brazilian National Food and Nutrition Surveillance System.

### Case study 2: the Brazilian Strategy for Breastfeeding and Complementary Feeding Promotion

The EAAB is the latest initiative of the Brazilian Ministry of Health focused on infant feeding. Rooted in the critical-reflexive concept, the EAAB aims to strengthen actions to promote, protect, and support breastfeeding and healthy complementary feeding for children under two years of age, improving the skills and abilities of health professionals within the scope of primary care ^57^ ^,^ ^58^ ^,^ ^59^. The blueprint for implementing the EAAB outlines a training cascade for health professionals, starting at the federal level, down to the states and municipalities. In the municipalities, “tutors of the EAAB” are trained to support continuing education activities (i.e., workshops) with primary health providers. Primary health clinics must comply with the following six quality criteria (core functions of the EAAB) to be certified in the EAAB by the Brazilian Ministry of Health: (1) develop systematic individual or collective actions to promote breastfeeding and healthy complementary feeding; (2) monitor breastfeeding and complementary feeding rates; (3) have an instrument for organizing child health care (flowchart, map, protocol, line of care, or other) to detect problems related to breastfeeding and healthy complementary feeding; (4) comply with the NBCAL and *Law n. 11,265/2006* and do not distribute breast milk “substitutes” in the primary health clinics; (5) participation of at least 85% of the primary care professionals in the continuing education workshop; (6) comply with at least one activity to encourage breastfeeding, and one for healthy complementary feeding agreed in the action plan ^60^. 

## Using implementation science to achieve adequate nutrition by 2030

Our implementation analysis identified essential elements across RE-AIM dimensions that could improve the implementation and sustainability of both programs and practices. Documenting and paying attention to the RE-AIM elements have been proved to increase the likelihood of improving successful implementation and, ultimately, the health of the entire population ^49^. Furthermore, it highlights a profound common challenge of implementing child nutrition actions in Brazil: how to implement at scale while maintaining effectiveness and sustainability over time? This dilemma underlies the growing recognition of the critical importance of addressing the “implementation gap” ^61^, which has stimulated interest in developing and applying implementation science in nutrition.

Implementation science in nutrition is a body of systematized knowledge about how to improve the implementation of nutrition-specific and sensitive interventions ^61^ ^,^ ^62^. It is not enough to know that a nutrition intervention is efficacious; it is also necessary to understand how to identify barriers, build upon strengths, and address weaknesses of actions in real-world conditions. Implementation science in nutrition can help understand how to effectively scale up an intervention to help improve nutrition ^62^. Implementing at scale is a central theme for successful compliance with the SDGs by 2030 ^63^ ^,^ ^64^. Implementation at scale refers to planned efforts to scale up or expand nutrition actions with proven effectiveness for large segments of the target population, promoting sustainable policies and programs ^63^ ^,^ ^64^. A systematic review identified nine key elements for successful scale implementation of nutrition programs ^65^: (1) having a clear vision or goal for impact; (2) understanding clearly intervention characteristics and expected impacts; (3) having an enabling organizational context for scaling up; (4) establishing drivers such as catalysts, champions, systemwide ownership, and incentives; (5) choosing contextually relevant strategies and pathways for scaling up; (6) building operational and strategic capacities; (7) ensuring the adequacy, stability, and flexibility of financing; (8) ensuring adequate governance structures and systems; and (9) embedding mechanisms for monitoring, learning, and accountability. Therefore, using implementation research and frameworks for understanding and intervening in these key elements is critical to advance the scale-up and sustainability of nutrition policies and programs in Brazil ^63^.

## Final considerations

In the last 30 years, Brazil has achieved nearly universal access to primary health care for the population, thanks to the expansion of the coverage of the Family Health Strategy, which reaches 60% of the population ^66^. This context has enabled and facilitated the implementation of breastfeeding and malnutrition strategies and programs, and it has certainly contributed to improving several indicators of infant and young nutrition outcomes ^12^ ^,^ ^67^ ^,^ ^68^. Our analysis indicated: (1) a shift in the paradigm of malnutrition programs, with investment in income transfer programs; (2) efforts to implement high coverage specific micronutrient supplementation programs; (3) a broad implementation of actions to protect, promote, and support breastfeeding and complementary feeding, aligned with conceptual models of determinants; and (4) recent efforts aimed at combating childhood obesity, in line with guidelines aimed at promoting healthy environments and intersectoral actions. These efforts have great potential to advance infant and young children’s nutrition indicators in Brazil.

However, fiscal policies implemented in 2016 ushered in austerity measures that, alongside the current Brazilian government’s new environmental, educational, and health policy, could reverse the hard-earned achievements of the SUS, impacting the implementation of infant feeding and nutrition policies ^69^. Therefore, understanding the trajectory of implementation of these policies allows us to extract the lessons necessary to face the challenges that arise. From the point of view of theoretical models, the UNICEF Nutrition Strategy 2020-2030 updates the conceptual framework on the determinants of maternal and child nutrition ^70^. Using a positive narrative about what contributes to good nutrition in children and women, the framework provides conceptual clarity on the enabling determinants (governance, resources, and positive social and cultural norms), underlying determinants (food, feeding - including age-appropriate dietary practices and responsive feeding - and healthy food environments), and immediate determinants (good diets - driven by adequate food and feeding for children and women - and good care - driven by adequate services and practices for the care of children and women). It also presents the vertical and horizontal interconnectedness of all determinants and their impact on the positive survival, growth, development, performance, and economic outcomes resulting from improved maternal and child nutrition ^70^.

Although improving infant and young children’s nutrition requires information about biology and epidemiology, the increasing complexity and multisectorality of nutrition initiatives highlight sociopolitical factors that determine which actions are appropriate and acceptable ^61^ ^,^ ^62^. With this, there is a growing need for information across socioecological domains to determine how best to design and implement intended activities to achieve the desired changes ^61^ ^,^ ^62^. Therefore, the adoption of this model in the implementation of national infant feeding policies can help in the recognition that biology and epidemiology are important in defining strategies. However, acknowledging that food and nutrition are a complex sociopolitical field requires action throughout all layers of social determinants of child food and nutrition, in addition to indicating ways to strengthen intersectoral policies ^61^ ^,^ ^62^ ^,^ ^71^.

The exercise of applying the RE-AIM, one of the most widespread frameworks in implementation research, in the analysis of two brief case studies on breastfeeding policies in Brazil, allowed us to identify implementation barriers in different dimensions. We found common barriers related to (1) unclear goals regarding the reach of programs; (2) challenges in assessing effectiveness and fidelity/quality during the real-world implementation; (3) discontinuation or lack of funding and lack of monitoring and evaluation, impacting the sustainability of programs. Therefore, implementation research can provide pragmatic programmatic lessons and guidance on how to scale up nutrition interventions by better understanding different contexts, identifying barriers and facilitators, and allocating resources and funds for maximum impact ^72^. Evidence shows that advocacy is needed to generate the necessary political will to enact legislation and policies to protect, promote, and support breastfeeding ^43^.

Hence, high-quality implementation research within large-scale nutrition programs must become a priority to catalyze progress. Implementation research for nutrition is not new but has not been prioritized ^71^. In fact, some steps are being taken to strengthen and expand the EAAB based on implementation science. Two studies used program impact pathway analysis to document the implementation as well as barriers and facilitators to scale up the EAAB in Brazil ^57^ ^,^ ^58^. With the support of the Brazilian Ministry of Health, the results of these studies are being incorporated into meetings to support EAAB managers at the federal, state, and municipal levels. In addition, the tutor training curriculum was revised and included the RE-AIM dimensions to support tutors in implementing the EAAB at the local level ^73^. Hopefully, experiences of this kind can be extended to the effective scale-up and sustainability of infant and young child nutrition policies and help Brazil in achieving adequate nutrition for the 2030 SDGs.
